# Water as an agent for the morphology modification of metal oxalate materials on the nanoscale: from sheets to rods

**DOI:** 10.1038/srep19282

**Published:** 2016-01-14

**Authors:** Minog Kim, YooJin Kim, WonJong Kwon, Sungho Yoon

**Affiliations:** 1 Department of Bio & Nano Chemistry, Kookmin University, 861-1 Jeongneung-dong, Seongbuk-gu, Seoul 136-702, Republic of Korea; 2 Engineering Ceramic Center, Korea Institute of Ceramic Engineering and Technology, Icheon 467-843, Republic of Korea; 3 LG Chem Research Park, 104-1 Moonji-dong, Yuseong-gu Daejeon 305-380, Republic of Korea

## Abstract

A number of approaches have been used to control the shape of metal oxalates, which often used as precursors for metal oxide nanomaterials. However, attempts to use water as a regulator have not been reported. Here in we report systematic studies on related topics: nanosheets, composed of 1-dimensional [M(C_2_O_4_)(EG)] (M = Zn or Co) polymeric structure, could be transformed into nanorods by using water as a shape-shifting agent because water can readily substitute EG ligand, leading alternation of inter-chain hydrogen bonding interactions. In addition, heat-treatment of these nanomaterials with diverse morphologies resulted in porous metal oxides with high degrees of shape retention.

Transition metal oxides containing ZnO and Co_3_O_4_ have recently attracted much attention for possible applications in pigments^1^, gas sensing^2^,^3^,^4^, catalysis^4^,^5^, sensors^6^,^7^, supercapacitors^8^,^9^, and Li-ion batteries^10^,^11^. Because the nanoscale morphology of metal oxides alters the physical and chemical properties of bulk materials, facile synthesis methods that control crystal architecture are of great interest. Precursors for transition metal oxide have been continuously studied because they can often be tailored structurally; certain precursors are known to transform into the corresponding metal oxides with high degrees of shape retention or similarity. Transition metal oxalates, where oxalate (C_2_O_4_) is the simplest dicarboxylate, are representative of transition metal oxide precursors because of the advantages of low cost, various preparation methods, and easy transformation at relatively low temperatures^12^,^13^.

A number of approaches, including etching, solid-state reactions, solvothermal reactions and reverse-micelle processing, have been used to control the shape of metal oxalate precursors^13^,^14^,^15^,^16^. The synthesis of metal oxalate nanowires by etching a metal foil or via solid-state reactions involving grinding in a mortar are well known to possess various limitations^14^,^15^. Similarly, although solvothermal reactions are generally facile methods for preparing nano- and micro-rods, this approach requires finely detailed adjustments to tune the morphology of the precursors^13^. The synthesis of metal oxalate nanorods through the reverse-micelle process utilized surfactants, including cetyltrimethylammonium bromide, which resulted in the high cost and low yield of the process^16^. Liquid-phase reactions without complicated multistep routes are considered ideal methods for the preparation and alternation of metal oxalate precursors.

Several efforts in liquid-phase synthesis have been made to prepare metal oxalate precursors with diverse morphologies using water as a solvent; a water-controlled precipitation approach^12^ has been proposed to control the oxalate precursor’s shape by changing the solvents upon its synthesis, and studies on changing the shape of the precursor structure by regulating the ratio of a mixed solvent of water/ethylene glycol (EG; C_2_H_6_O_2_)^13^ have been reported. In addition, efforts to change the shape of oxalate materials by adjusting the ratio of water/ethanol mixed solvent have been attempted^17^. However, attempts to use water not as a solvent but as a regulator have not been reported; studies that clearly described the role of water in the adjustment of the shape of the precursor structure are rare, and no systematic approach to examine water as a regulator has been attempted. Herein, we report a first attempt to control Zn(II) or Co(II) oxalate nanostructures by a simple adjustment of the water content in the preparation steps of formation and isolation, resulting in spectrally observable changes in morphology.

The single-crystal structure of [Zn(C_2_O_4_)(EG)] (**1**), a derivative of the zinc oxalate compound, has been recently reported^18^. Block crystals of **1** with dimensions of 0.26 × 0.25 × 0.24 mm were obtained from a reaction solution of Zn^2+^ ions and EG in H_2_SO_4_ using an autoclave, which created high pressures and elevated temperatures. The oxidation of EG in aqueous H_2_SO_4_ generated oxalic acid, which subsequently ligated to Zn^2+^ resulting in **1**. In the reported X-ray structure of **1**, the Zn^2+^ ion had a distorted octahedral geometry in which the two O atoms of the EG ligand were coordinated to one Zn^2+^ ion (Fig. S1a). The dicarboxylated oxalate ligand connects two Zn^2+^ ions to form a one-dimensional zigzag polymeric structure (Fig. 1a). Inter-polymeric chain hydrogen bonding (H-bonding) interactions between the O atoms of the oxalates and the H-O groups of the EGs organized the one-dimensional polymer into two-dimensional layers in the *bc* plane, as shown in Fig. S1b. The two CH_2_ units in the EG ligands, oriented perpendicularly above and below the plane, interdigitated and became stacked in an orderly fashion to form a three-dimensional structure (Figs 1b and S1c). Because the van-der waals force between the *bc* planes is relatively weak, we hypothesized that compound **1** could be prepared with a two-dimensional nanosheet morphology rather than a micro-sized cubic structure by simply varying the synthetic route through the use of an oxalate, EG, and Zn^2+^ ions under mild conditions.

The reaction of oxalic acid and zinc sulfate in a 1:1 molar ratio in EG, followed by washing with anhydrous tetrahydrofuran (≥99.9%), precipitated a white solid with excellent dispersion properties. Through scanning electron microscopy (SEM), thin nanosheets with approximate dimensions of 1 μm × 2 μm × 30 nm can be observed (Fig. 1c). Nanosheet of **1** is characterized using diverse analyses. Peaks observed at 1620 (C = O), 1364 and 1319 (O-C = O), 820 (C-C), and 494 cm^−1^ (Zn-O) indicate the presence of zinc oxalate, and those at 3200 (O-H), 1471 (C-H), and 1065 and 1032 cm^−1^ (O-C) correspond to the EG ligand (Fourier-transform infrared spectrum, Fig. S2). In addition, the ratio of Zn:oxalate:EG can be quantitatively confirmed as 1:1:1 via thermogravimetric analysis (TGA) (Fig. S3). The XRD pattern observed for the nanosheets of **1** is well matched with the anticipated peaks, which ware generated by conversion of the single-crystal data of **1** (Fig. 1d)^18^. Meanwhile, the phenomenon of broadening observed in the XRD peaks from the nanosheets synthesized here compared to those in the reference reiterates that **1** is likely to be a nano-, rather than micro-sized, structure. To confirm these speculation, the transmission electron microscopy (TEM) image was obtained, which showed that **1** synthesized here consists of several interleaved nanosheets (Fig. S4).

Upon indexing the grid included in the TEM image, most of the grid was observed at low-intensity peaks in the 2θ range of 35°–45° in the X-ray diffraction(XRD) patterns (Fig. 1d,e). The geometric structure of **1**, obtained in Fig. 1e by fast-Fourier-transform TEM (FFT-TEM) and high-resolution TEM (HR-TEM), corresponds with that represented schematically in Fig. 1f. This shows that the nano- sheet is made by inter-chain H-bonding interactions between EG and oxalate in the different zigzag chains, as shown in Fig. S1b. In addition, EG is widely known to act as a chelating agent and has been used in metal oxalate studies to extend the lengths of rod-like structures or assist the structural growth of complexes in a one-dimensional fashion^13^,^19^,^20^,^21^. Interestingly, however, we found that EG does not help **1** to grow lengthwise, but instead helps the structure to grow two-dimensionally in sheets.

EG in nanosheet of **1** may be substituted by other ligands such as OH_2_ and the inter-chain H-bonding interactions, which are driving forces of the formation of nanosheet, can be expected to be perturbed easily. We hypothesized that the nanosheet morphology of **1** can be altered by treating water. The as-synthesized **1** was washed with 94% ethanol in water, and a well-dispersed white precipitate was obtained. In this case, SEM imaging surprisingly reveals an aggregation of rods (600 × 35 × 30 nm) (Figs 2a,b and S5), instead of sheets. In the FT-IR spectrum of the nanorods, in contrast to that from the nanosheets, peaks at 3400 cm^−1^ corresponding to OH_2_ and 3200 cm^−1^ corresponding to EG were observed simultaneously, indicating that the rod had the structural formula [Zn(C_2_O_4_)(EG)_x_(OH_2_)_y_] (Fig. S2). By quantitative TGA, OH_2_ and EG were determined to be coordinated to zinc oxalate in a 1:1 ratio (Fig. S6). Based on these results, it can be surmised that the EG ligand coordinated to the Zn^2+^ ion may be substituted by water from the solvent, creating rods that torn away from the sheets. With additional water treatment, all the EG ligands are replaced by water to form the well-known compound of zinc oxalate dihydrate, while maintaining the rod-shaped morphology of the material (Figs S2 and S7). The XRD patterns for the completely water-substituted rod-shaped [Zn(C_2_O_4_)(OH_2_)_2_] clearly differ from that of [Zn(C_2_O_4_)(EG)] (Fig. 2c), and is well matched with the XRD data of referenced micro-sized [Zn(C_2_O_4_)(OH_2_)_2_] (JCPDS No. 25-1029)^22^. As the EG ligands are replaced by water, the intensity peaks near 2θ = 40° in **1** (Fig. 1d) disappear and a new peak near 2θ = 35° is observed. This peak is also observed by TEM. The d value of the lattice at the edges of the split rod (Fig. 2d,e) is consistent with the peak near 2θ = 35° in the XRD pattern. The corresponding geometric structure of [Zn(C_2_O_4_)(OH_2_)_2_] is depicted in Fig. 2f, showing that the surface of the nanorod is made by H-bonding between OH_2_ and oxalate (Fig. S8). This indicates that alternations of the H-bonding in the surface of the nanosheet cause the division of the nanosheet into nanorods, in accordance with the change of ligand species from EG to OH_2_.

Figure 3 shows that increasing the proportion of water in the washing solvent leads to the gradual substitution of EG by water, resulting in the tearing of the nanosheets into nanorods. By changing the ligand, the orthorhombic unit cell of [Zn(C_2_O_4_)(EG)] is transformed to the monoclinic form of [Zn(C_2_O_4_)(OH_2_)_2_]. Furthermore, in Figs 1f and 2f, it was confirmed that the structures of the nanosheet and the nanorod are composed of hydrogen bonds between the ligand species of EG and OH_2_ and oxalate, respectively. Notably, the average hydrogen bond lengths in [Zn(C_2_O_4_)(EG)] and [Zn(C_2_O_4_)(OH_2_)_2_] are 1.90 and 2.05 Å, respectively, indicating that the H-bonding interactions weaken as [Zn(C_2_O_4_)(EG)] changes to [Zn(C_2_O_4_)(OH_2_)_2_] (Table S1)^18^,^23^,^24^. This may facilitate the formation of crack in the structure, which would be consistent with a nanostructured sheet that splits into rods. This phenomenon occurs because water acts as a good coordinating ligand, capable of binding to the Zn^2+^ ions.

This interaction of water with the metal ions is not limited to Zn^2+^ and may be applied to other divalent metal cations. With this assumption, we tested whether a similar phenomenon occurs in Co(II) oxalate. Upon synthesizing [Co(C_2_O_4_)(EG)] in the same manner as we synthesized [Zn(C_2_O_4_)(EG)], a pink precipitate in the shape of nanosheets is obtained (Figs 4a and S9). Figures S10–S12 show that the XRD, FT-IR and TGA analysis of [Co(C_2_O_4_)(EG)] closely resemble that of the **1** nanosheet. In XRD patterns (Fig. S10), several peaks with different intensities seem to be caused by different crystal sizes, in accordance with the change of the metal ion^25^. In the same way, nanorod-shaped [Co(C_2_O_4_)(EG)_x_(OH_2_)_y_] and [Co(C_2_O_4_)(OH_2_)_2_] were successfully synthesized (Figs 4b,c, and S13). With the help of various analyses, it was confirmed that EG displays the same phenomenon with Co^2+^ as it does with Zn^2+^ when the ligand is gradually substituted with OH_2_ (Figs S11, S14–16). In addition, through TEM analysis, we confirmed that nanosheet-shaped [Co(C_2_O_4_)(EG)] and nanorod-shaped [Co(C_2_O_4_)(OH_2_)_2_] have the same plane configurations respectively, as those shown in Figs 1f and 2f (Figs S17 and S18). Therefore, similarly to the Zn^2+^ complex, the Co^2+^ complex also forms a nanosheet that is divided into rods by altering the hydrogen bond interactions between the oxalate and the corresponding ligands (EG or OH_2_). In conclusion, [Co(C_2_O_4_)(EG)] varies its shape in accordance with a mechanism very similar to that dictating the shape of [Zn(C_2_O_4_)(EG)], and it was found that the morphology was driven by the water content during the reaction.

In order to confirm the applicability of the substances as precursors to metal oxides, the as-prepared samples with diverse morphologies were heated for 2 h at 500 °C under air. The materials were successfully converted to porous ZnO or Co_3_O_4_, which maintained the shapes of each precursor (Figs S19 and 20). For characterization of the textural properties of the as-synthesized ZnO and Co_3_O_4_ samples, BET (Brunauer-Emmett-Teller) gas-sorption measurements were performed. As shown in Fig. S21, the nitrogen adsorption-desorption isotherms with hysteresis loops belong to typical type IV, indicating the ZnO and Co_3_O_4_ samples have mesoporous structures. The BET specific surface area of these samples are measure to be 47, 56, 56 and 47 m^2^g^−1^ for nanosheet-shaped ZnO, nanorod-shaped ZnO, nanosheet-shaped Co_3_O_4_ and nanorod-shaped Co_3_O_4_, respectively, which reveals that all of them possess relatively high specific surface areas and may exhibit potential applications in catalysis, sensing and so on.

In conclusion, we synthesized a [Zn(C_2_O_4_)(EG)] compound with a nanosheet morphology, which is split into rods by the gradual substitution of the EG ligands with water. The same mechanism was also observed with Co^2+^. Thereafter, it was possible to convert the Zn(II) or Co(II) oxalate precursors with various morphologies to porous ZnO or Co_3_O_4_ materials while maintaining the water-modulated architectures by heat treatment. Although efforts to tailor the shapes of metal oxalate precursors continue, this study is the first we know of that describes gradual changes in the metal oxalate precursor morphology as a function of water content during the liquid-phase synthesis. This mechanism could be developed into a general method to ensure that the same mechanism applies to all transition metals; this study is currently underway.

## Additional Information

**How to cite this article**: Kim, M. *et al*. Water as an agent for the morphology modification of metal oxalate materials on the nanoscale: from sheets to rods. *Sci. Rep.* **6**, 19282; doi: 10.1038/srep19282 (2016).

## Supplementary Material

Supplementary Information

## Figures and Tables

**Figure 1:**
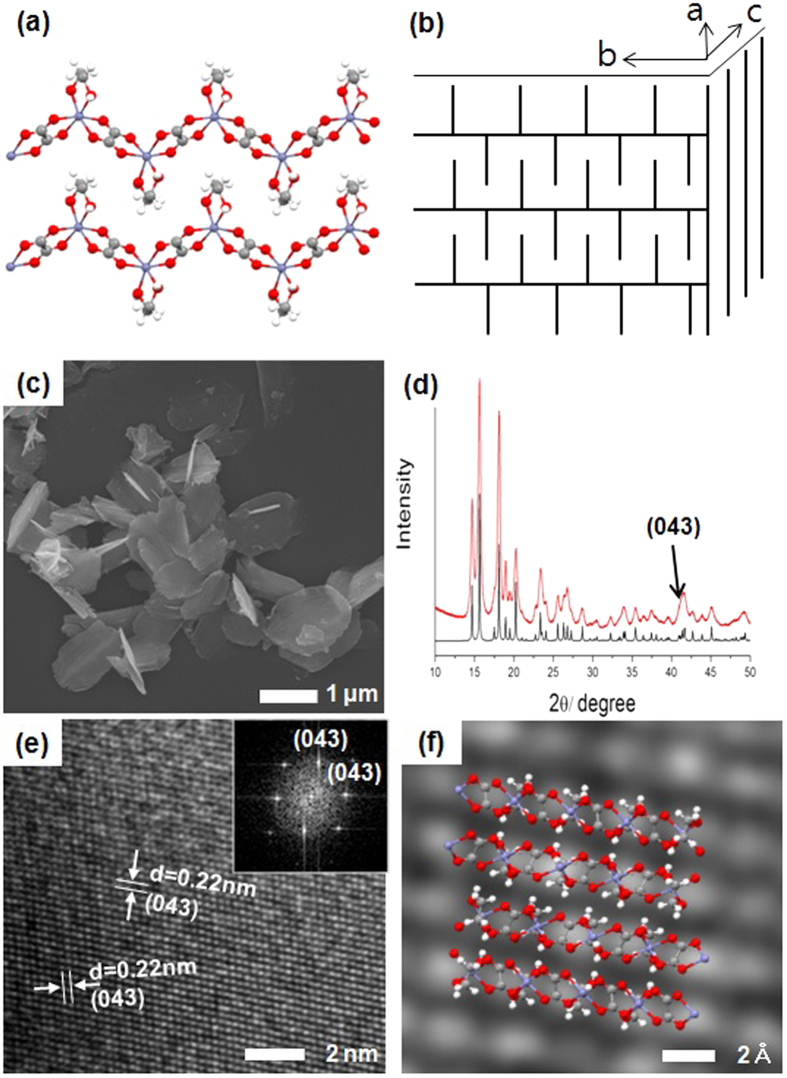
Illustrations of the nanosheet structure of 1: (**a**) one-dimensional zigzag polymeric structure (white: hydrogen, gray: carbon, red: oxygen, and blue: zinc), (**b**) three-dimensional structure. (**c**) SEM image, (**d**) XRD patterns (black: XRD spectrum deduced from single-crystal X-ray diffraction data of 1, red: as-synthesized nanosheet of 1), (**e**) high-resolution and (inset) fast-Fourier-transform transmission electron microscopy (HR- and FFT-TEM), and (**f**) corresponding geometric structure of 1 (white: hydrogen, gray: carbon, red: oxygen, and blue: zinc).

**Figure 2:**
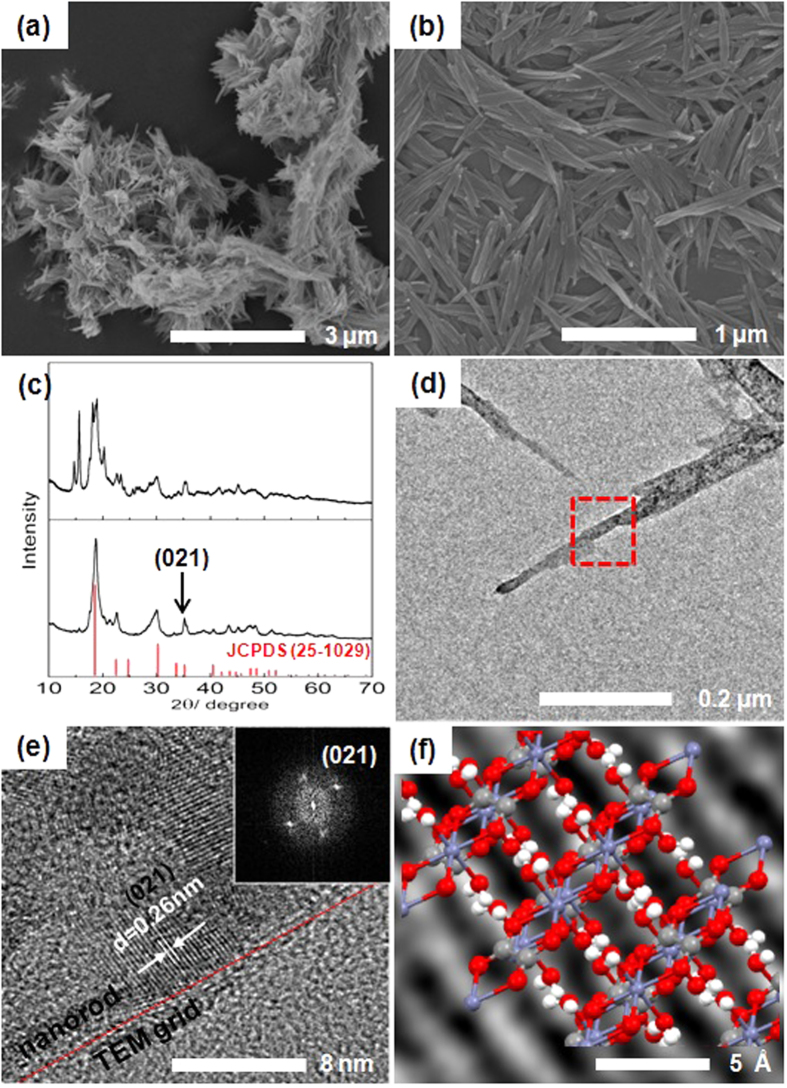
(**a,b**) SEM images of rod-shaped [Zn(C_2_O_4_)(EG)_x_(OH_2_)_y_], (**c**) XRD patterns of nanorod-shaped [Zn(C_2_O_4_)(EG)_x_(OH_2_)_y_] (top) and [Zn(C_2_O_4_)(OH_2_)_2_] (bottom). (**d**) HR-TEM, (**e**) HR-TEM, and (inset) FFT images of rod-shaped [Zn(C_2_O_4_)(OH_2_)_2_], and (**f**) corresponding geometric structure of [Zn(C_2_O_4_)(OH_2_)_2_] (white: hydrogen, gray: carbon, red: oxygen, and blue: zinc).

**Figure 3:**
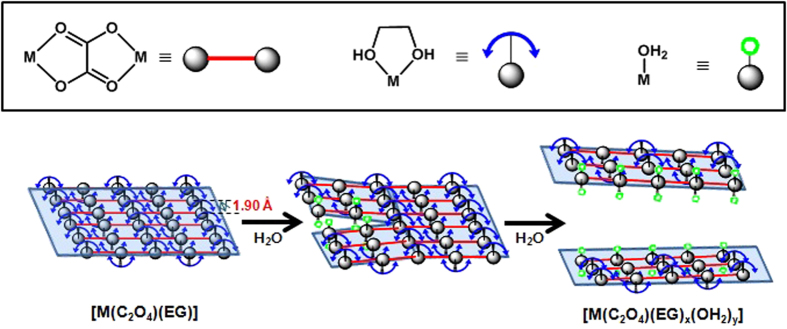
Substitution of the ligands in the nanostructure corresponding to the polymeric structure.

**Figure 4:**
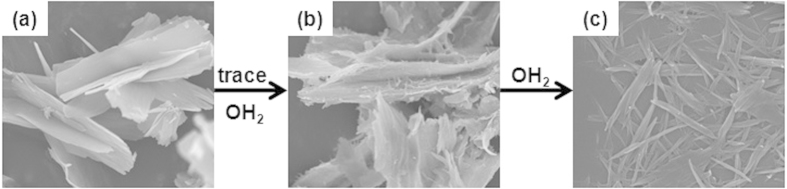
Substitution of ligands in the [Co(C_2_O_4_)(EG)] complex. (**a**) nanosheet-shaped [Co(C_2_O_4_)(EG)], (**b**) nanorod-shaped [Co(C_2_O_4_)(EG)_x_(OH_2_)_y_], and (**c**) nanorod-shaped [Co(C_2_O_4_)(OH_2_)_2_].
