# Supplementary material for: Water as an agent for the morphology modification of metal oxalate materials on the nanoscale: from sheets to rods
Source: Sci Rep. 2016 Jan 14;6:19282. doi: 10.1038/srep19282 (PMC4725861; doi:10.1038/srep19282)
Supplement: Supplementary Information [file srep19282-s1.pdf]

Supporting Information

Water as an agent for the morphology modification of metal oxala te materials on the nanoscale: from sheets to rods

Minog Kim,[1] YooJin Kim,[2] Won Jong Kwon[3] and Sungho Yoon,[1,*]

Contents

I. Zinc oxalate (ZnC_2O_4) precursors

A. Experimental information

B. X-ray structure of $[Zn(C_2O_4)(EG)]$ compound **(1)**

C. FT-IR spectra of the synthesized-samples

D. TGA-DTA analysis of nanosheet-shaped $[Zn(C_2O_4)(EG)]$ complex

E. HR-TEM images of nanosheet-shaped $[Zn(C_2O_4)(EG)]$ complex

F. AFM analysis of nanorod-shaped $[Zn(C_2O_4)(OH_2)_2]$ complex

G. TGA-DTA analysis of nanorod-shaped $[Zn(C_2O_4)(EG)_x(OH_2)_y)]$ complex

H. TGA-DTA analysis of nanorod-shaped $[Zn(C_2O_4)(OH_2)_2]$ complex

I. The corresponding geometric structure of nanorod-shaped $[Zn(C_2O_4)(OH_2)_2]$ complex

J. Change of Zn-O distance, hydrogen bond distance and hydrogen bond angle

II. Cobalt oxalate (CoC_2O_4) precursors

K. Experimental information

L. SEM, XRD and TGA analyses of $[Co(C_2O_4)(EG)]$ complex and FT-IR spectra of the synthesized-samples

M. SEM, XRD, HR-TEM, FTT and TGA analyses of nanorod-shaped $[Co(C_2O_4)(EG)_x(OH_2)_y]$ and nanorod-shaped $[Co(C_2O_4)(OH_2)_2]$

III. ZnO and Co_3O_4

N. SEM, HR-TEM, FTT images and XRD graphs of the prepared-ZnO samples

O. SEM, HR-TEM, FTT images and XRD graphs of the prepared-Co_3O_4

A. Experimental information

Materials

Oxalic acid (Aldrich, ≥99.0%), zinc sulfate heptahydrate (Aldrich, ≥99.0%) and cobalt sulfate heptahydrate (Aldrich, ≥99.0%) were dehydrated at 200℃ under vacuum overnight. Anhydrous ethylene glycol (Aldrich, 99.8%) and anhydrous tetrahydrofuran (Aldrich, ≥99.9%) were used as received without further purification. Water was distilled twice. Molecular sieves (3 Å, Aldrich) were washed with acetone and vacuum-dried at 200℃ before use. Ethanol (Daejung, 94%) was used.

Synthesis of nanosheet-shaped [Zn(C_2O_4)(EG)] complex (1)

Oxalic acid (1.00 mmol, $H_2C_2O_4$) was dissolved in ethylene glycol (15 mL), followed by the addition of zinc sulfate (1.00 mmol, $ZnSO_4$) and ca. 0.70 g 3Å molecular sieves. After stirring for 2 h, the white precipitate was washed with 33 mL of anhydrous tetrahydrofuran (THF) and isolated by centrifugation for 3 times (≤ 0.48 mmol of water). The obtained powder was dried in a vacuum oven at 60 ℃ overnight.

* Anhydrous THF was used in the consideration of the tough nature that azeotropic mixture is hard to be separated from water, even though the same phenomenon was observed upon using anhydrous ethanol. As a result of the study, it was observed that solvents with water excluded as much as possible, such as anhydrous methanol, anhydrous ethanol, and anhydrous THF, bring the same result.

Synthesis of nanorod-shaped [Zn(C_2O_4)(EG)$_x$(OH_2)$_y$] complex

Oxalic acid (1.00 mmol, $H_2C_2O_4$) was dissolved in ethylene glycol (15 mL), followed by the addition of zinc sulfate (1.00 mmol, $ZnSO_4$) and ca. 0.70 g 3Å molecular sieves. After stirring for 2 h, the white precipitate was washed with 33 mL of ethanol (94%) and isolated by for 3 times (≤ 260 mmol of water). The obtained powder was dried in a vacuum oven at 60 ℃ overnight.

Synthesis of nanorod-shaped [Zn(C_2O_4)(OH_2)$_2$] complex

Oxalic acid (1.00 mmol, $H_2C_2O_4$) was dissolved in ethylene glycol (15 mL), followed by the addition of zinc sulfate (1.00 mmol, $ZnSO_4$) and ca. 0.70 g 3Å molecular sieves. After stirring for 2 h, the

white precipitate was washed with 33 mL of ethanol (94%) and isolated by centrifugation for 4 times (≤ 350 mmol of water). The obtained powder was dried in a vacuum oven at 60 ℃ overnight.

Synthesis of porous ZnO structures

The as-prepared zinc oxalate structures were annealed in air at 500 ℃ for 2 h at a heating rate of 2 ℃/min for converting to the porous ZnO structures.

Characterization

The morphologies of the as-prepared products were characterized by field emission scanning electron microscopy (a Hitachi FE-SEM S-4800). The Fourier transform-infrared (FT-IR) spectra were measured on JASCO FT/IR 460 PLUS using the KBr pellet method at room temperature. Thermogravimetric analysis/differential thermal analysis (TGA/DTA) was performed on TA instruments STA-1500 under N_2 gas (100mL/min) from room temperature to 600 ℃ at a heating rate of 10 ℃/min. The thickness of nanorod-shaped $[Zn(C_2O_4)(OH_2)_2]$ was determined by atomic force microscopy (AFM) (Seiko instrument model SPA 400) operated in non-contact mode (Si Cantilever). High-resolution transmission electron microscopy (HRTEM) images were taken on JEOL JEM-2100F. The nanosheet-shaped $[Zn(C_2O_4)(EG)]$ powder particles were dispersed in THF (99%), dropped on an ultrathin holey carbon support film, and then dried in air. The rod-shaped $[Zn(C_2O_4)(OH_2)_2]$ powder particles were ultrasonically dispersed in ethanol, dropped on a copper grid and then dried in air. Crystal structures of the as-prepared products were characterized by powder X-ray diffraction (XRD) on a Bruker D8 Focus X-ray powder diffractometer using CuKα radiation. BET surface area and N_2 adsorption–desorption measurements were conducted at 77 K using an automated gas sorption system (Belsorp II mini, BEL Japan,Inc.,).

B. X-ray structure of [Zn(C_2O_4)(EG)] compound (1)

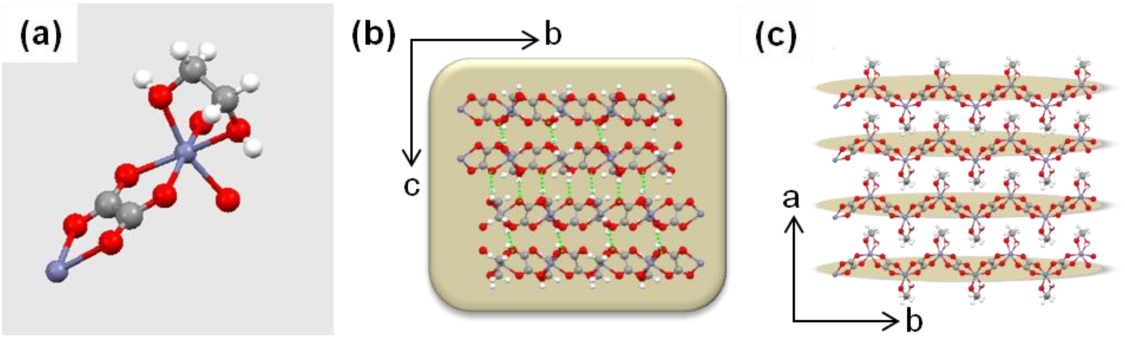

Fig. S1 Illustrated-structures of **1** having nanosheet shape (white: hydrogen, gray: carbon, red: oxygen, and blue: zinc): (a) single unit of [Zn(C_2O_4)(EG)], (b) *bc* plane (green-colored lines: H-bonding interactions) and (c) a*b* plane.

C. FT-IR spectra of the synthesized-samples

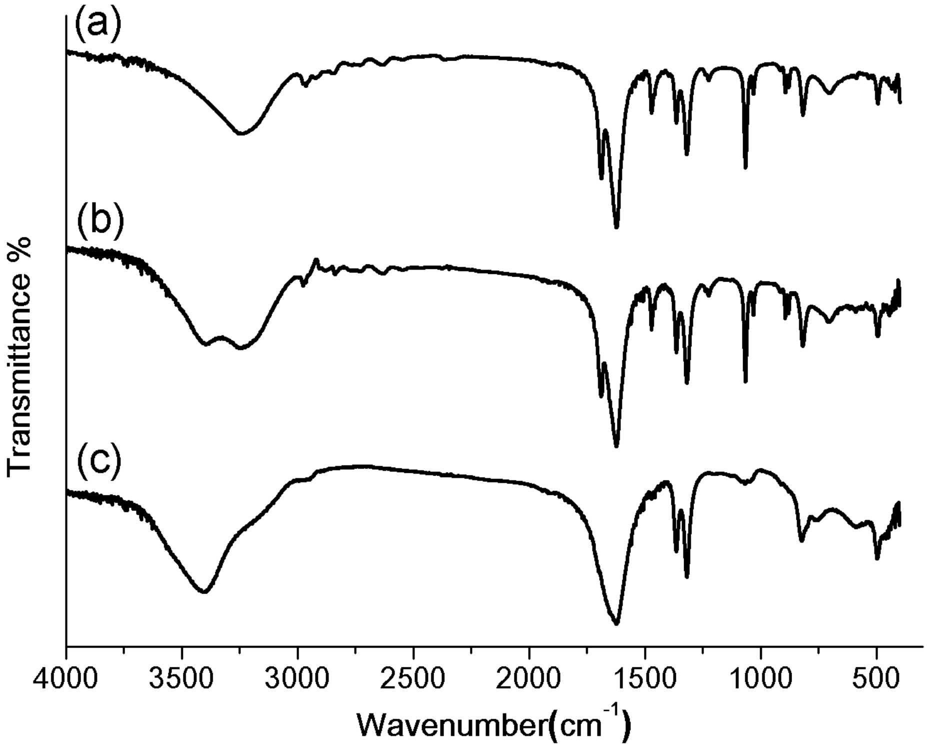

(a)	(b)	(c)	Assignment
	3393	3393	O-H stretch
3247	3247		
1687	1687		C=O stretch
1621	1621	1628	
1471	1471		C-H bend (alkane)
1364	1364	1363	symmetric and asymmetric O-C-O
1319	1319	1317	
1226	1226		non-identified
1065	1065		O-C stretch
1032	1032		
893	893		non-identified
878	878		non-identified
819	819	820	asymmetric O-C-O band
494	494	494	Zn-O stretch

Fig. S2 FT-IR spectra of (a) nanosheet-shaped $[Zn(C_2O_4)(EG)]$ complex, (b) nanorod-shaped $[Zn(C_2O_4)(EG)_x(OH_2)_y]$ complex and (c) nanorod-shaped $[Zn(C_2O_4)(OH_2)_2]$.

D. TGA-DTA analysis of nanosheet-shaped [Zn(C_2O_4)(EG)] complex

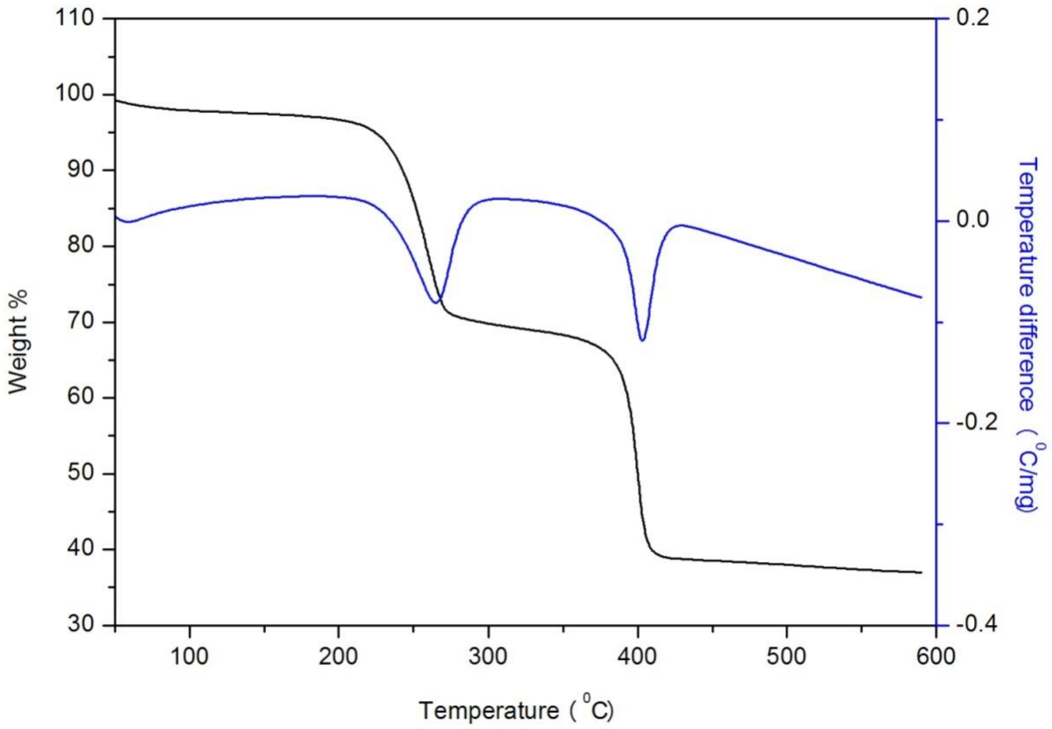

Temperature (℃)	Endothermic peak (℃)	Weight loss		Assginment
		Theoretical (%)	Observed (%)	
200~299	265	28.81	27.75	EG
345~440	403	33.42	32.19	CO, CO_2
		37.77	40.06	ZnO

Fig. S3 TGA-DTA analysis of nanosheet-shaped [Zn(C_2O_4)(EG)] complex (Black: TGA, Blue: DTA).

E. HR-TEM images of nanosheet-shaped [Zn(C_2O_4)(EG)] complex

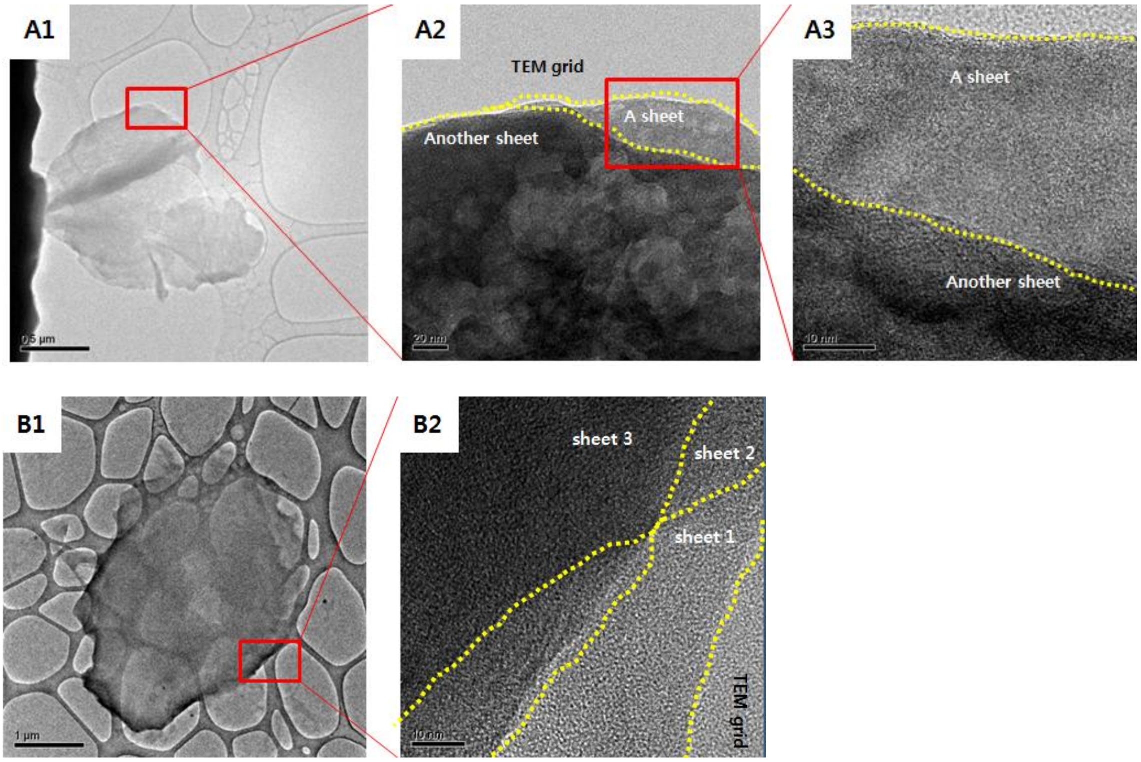

Fig. S4 High-resolution transmission electron microscopy (HRTEM) images of stacked several thin sheets consisting of [Zn(C_2O_4)(EG)].

F. AFM analysis of nanorod-shaped [$Zn(C_2O_4)(OH_2)_2$] complex

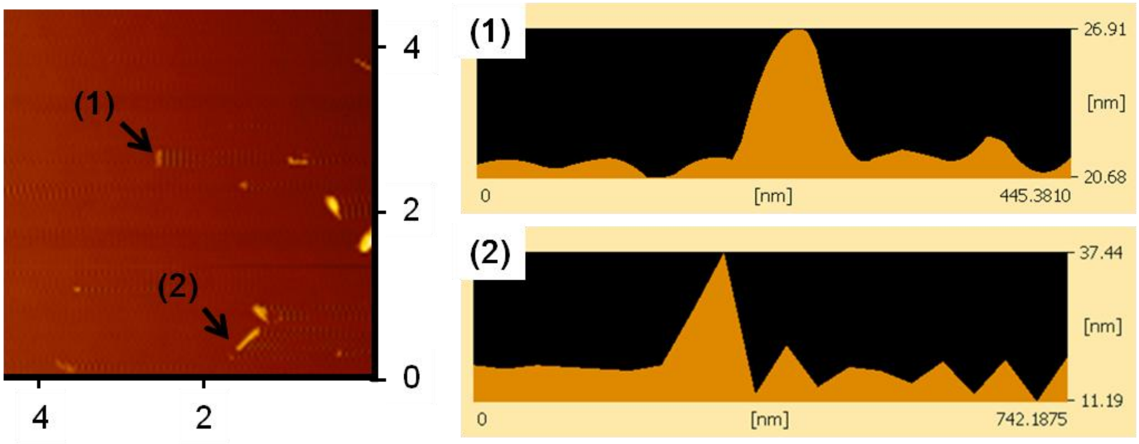

Fig. S5 Atomic-force microscopy (AFM) image and thickness profiles of nanorod-shaped [$Zn(C_2O_4)(OH_2)_2$] indicate that the average thickness is about 30 nm.

G. TGA-DTA analysis of nanorod-shaped [Zn(C_2O_4)(EG)$_x$(OH_2)$_y$)] complex

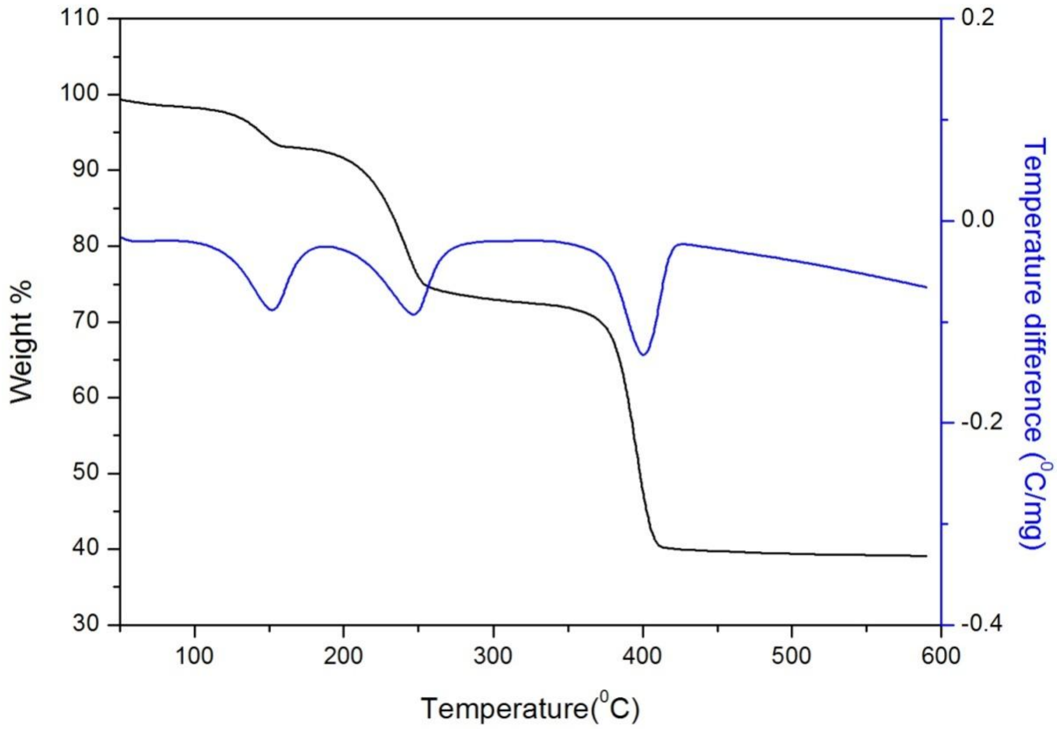

Temperature (℃)	Endothermic peak (℃)	Weight loss Observed (%)	Assginment
94~184	152	5.85	(OH_2)$_y$
184~291	246	19.68	(EG)$_x$
340~426	401	34.07	CO, CO_2
		40.39	ZnO

Fig. S6 TGA-DTA analysis of nanorod-shaped [Zn(C_2O_4)(EG)$_x$(OH_2)$_y$)] complex (Black: TGA, Blue: DTA).

H. TGA-DTA analysis of nanorod-shaped [Zn(C_2O_4)(OH_2)$_2$] complex

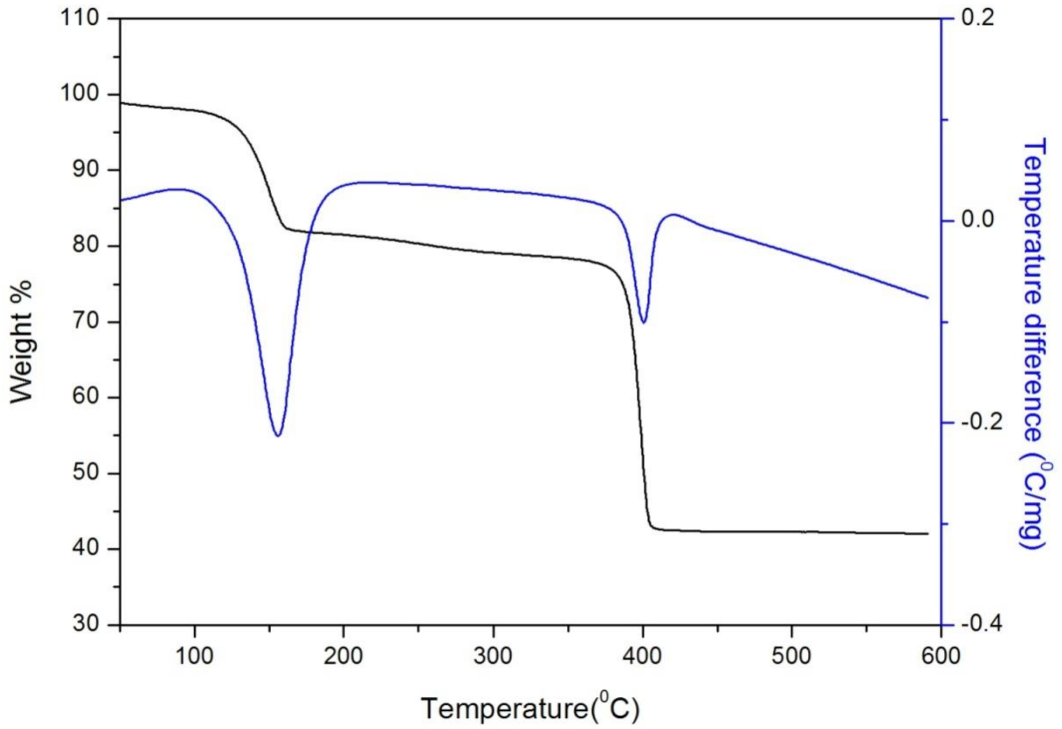

Temperature (℃)	Endothermic peak (℃)	Weight loss		Assginment
		Theoretical (%)	Observed (%)	
93~207	156	19.02	17.02	2(OH_2)
363~442	401	38.02	36.29	CO, CO_2
		42.96	42.49	ZnO

Fig. S7 TGA-DTA analysis of nanorod-shaped [Zn(C_2O_4)(OH_2)$_2$] complex (Black: TGA, Blue: DTA).

I. The corresponding geometric structure of nanorod-shaped $[Zn(C_2O_4)(OH_2)_2]$ complex

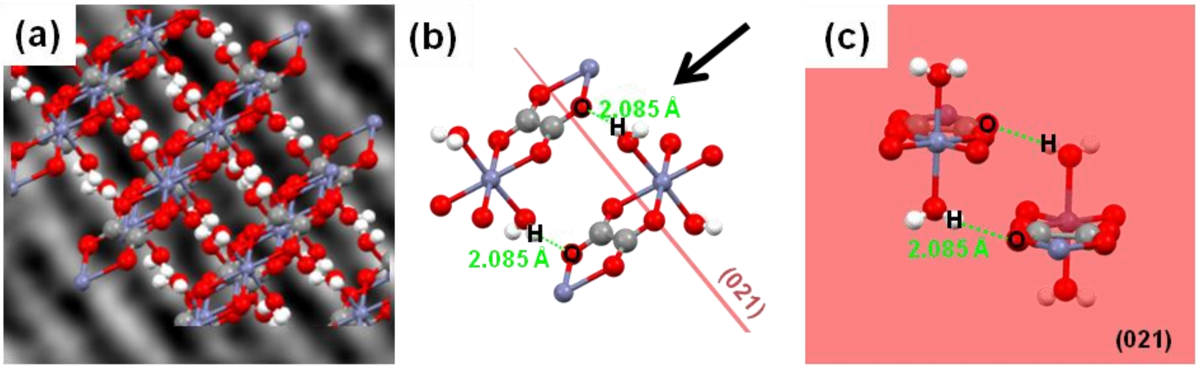

Fig. S8 (a) The geometric structure of $[Zn(C_2O_4)(OH_2)_2]$ corresponding with TEM image (Fig. 2f), (b) one repeating-unit of $[Zn(C_2O_4)(OH_2)_2]$; red line indicates the (021) face, and (c) when viewed from the black arrow direction of (b), two different $[Zn(C_2O_4)(OH_2)_2]$ chain interact with each other by H-bond.

J. Change of Zn-O distance, hydrogen bond distance and hydrogen bond angle

	$[Zn(C_2O_4)(EG)]^a$	$[Zn(C_2O_4)(OH_2)_2]^b$
Unit cell	Orthorhombic	Monoclinic
Zn-O (Å)	2.066(4)	2.118
O-H····O ^{i} (Å)	1.88(2)	2.008
O-H····O ^{i} (º)	170(7)	157.01
O-H····O ^{ii} (Å)	1.91(2)	2.085
O-H····O ^{ii} (º)	169(6)	158.78

[a] Data adapted from reference [S1], [b] Modified from reference [S2]

Table S1 Zn-O distance, hydrogen bond distances and the hydrogen bond angles caused by change of unit cell structure from orthorhombic to monoclinic.

K. Experimental information

Synthesis of nanosheet-shaped [Co(C_2O_4)(EG)] complex

Oxalic acid (1.00 mmol, $H_2C_2O_4$) was dissolved in ethylene glycol (15 mL), followed by the addition of cobalt sulfate (1.00 mmol, $CoSO_4$) and ca. 0.70 g 3Å molecular sieves. After stirring for 2 h, the white precipitate was washed with 33 mL of anhydrous tetrahydrofuran (THF) and isolated by centrifugation for 3 times (≤ 0.48 mmol of water). The obtained powder was dried in a vacuum oven at 60 ℃ overnight.

* Anhydrous THF was used in the consideration of the tough nature that azeotropic mixture is hard to be separated from water, even though the same phenomenon was observed upon using anhydrous ethanol. As a result of the study, it was observed that solvents with water excluded as much as possible, such as anhydrous methanol, anhydrous ethanol, and anhydrous THF, bring the same result.

Synthesis of nanorod-shaped [Co(C_2O_4)($EG)_x$($OH_2)_y$] complex

Oxalic acid (1.00 mmol, $H_2C_2O_4$) was dissolved in ethylene glycol (15 mL), followed by the addition of cobalt sulfate (1.00 mmol, $CoSO_4$) and ca. 0.70 g 3Å molecular sieves. After stirring for 2 h, the white precipitate was washed with 33 mL of ethanol (94%) and isolated by for 3 times (≤ 260 mmol of water). The obtained powder was dried in a vacuum oven at 60 ℃ overnight.

Synthesis of nanorod-shaped [Co(C_2O_4)($OH_2)_2$] complex

Oxalic acid (1.00 mmol, $H_2C_2O_4$) was dissolved in ethylene glycol (15 mL), followed by the addition of cobalt sulfate (1.00 mmol, $CoSO_4$) and ca. 0.70 g 3Å molecular sieves. After stirring for 2 h, the white precipitate was washed with 33 mL of ethanol (94%) and isolated by centrifugation for 4 times (≤ 350 mmol of water). The obtained powder was dried in a vacuum oven at 60 ℃ overnight.

Synthesis of porous Co_3O_4 structures

The as-prepared cobalt oxalate structures were annealed in air at 500 ℃ for 2 h at a heating rate of 2 ℃/min for converting to the porous Co_3O_4 structures.

L. SEM, XRD and TGA analyses of [Co(C_2O_4)(EG)] complex and FT-IR spectra of the synthesized-samples

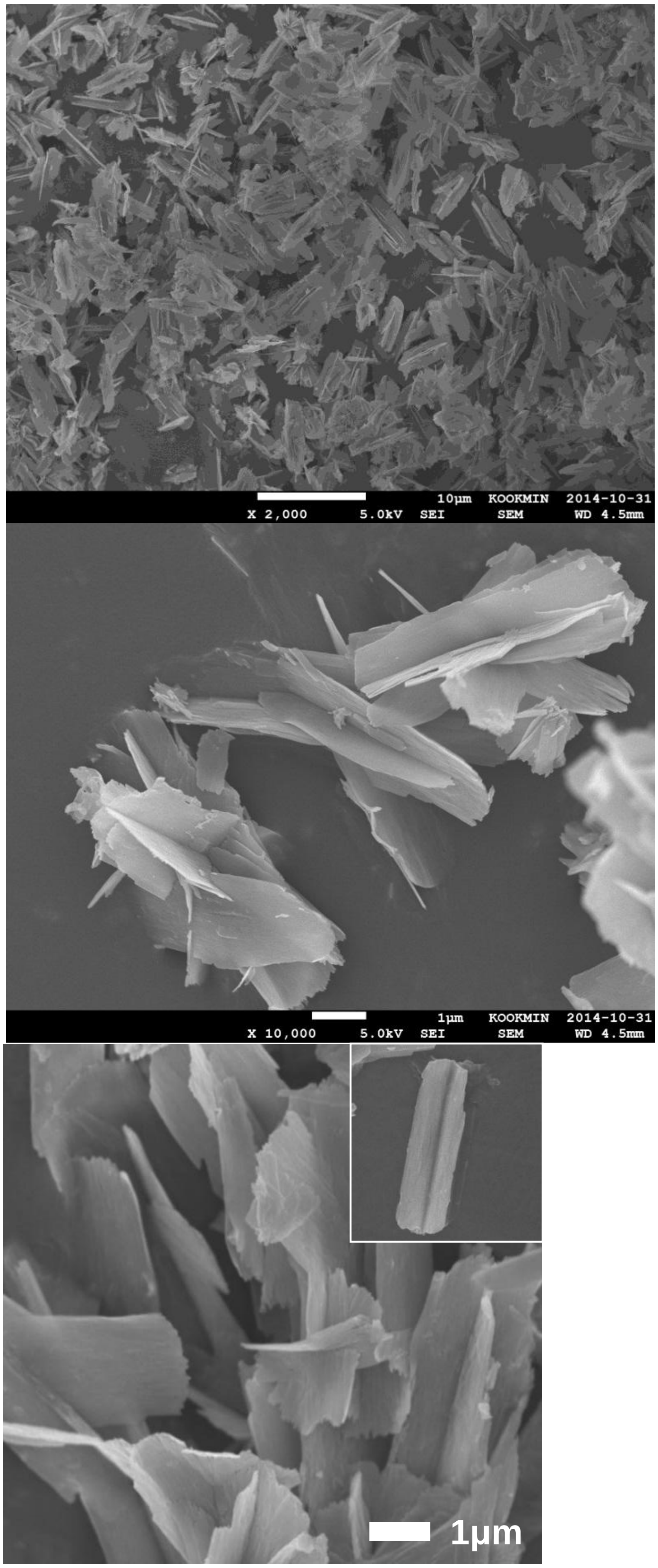

Fig. S9 SEM images of nanosheet-shaped [Co(C_2O_4)(EG)] complex.

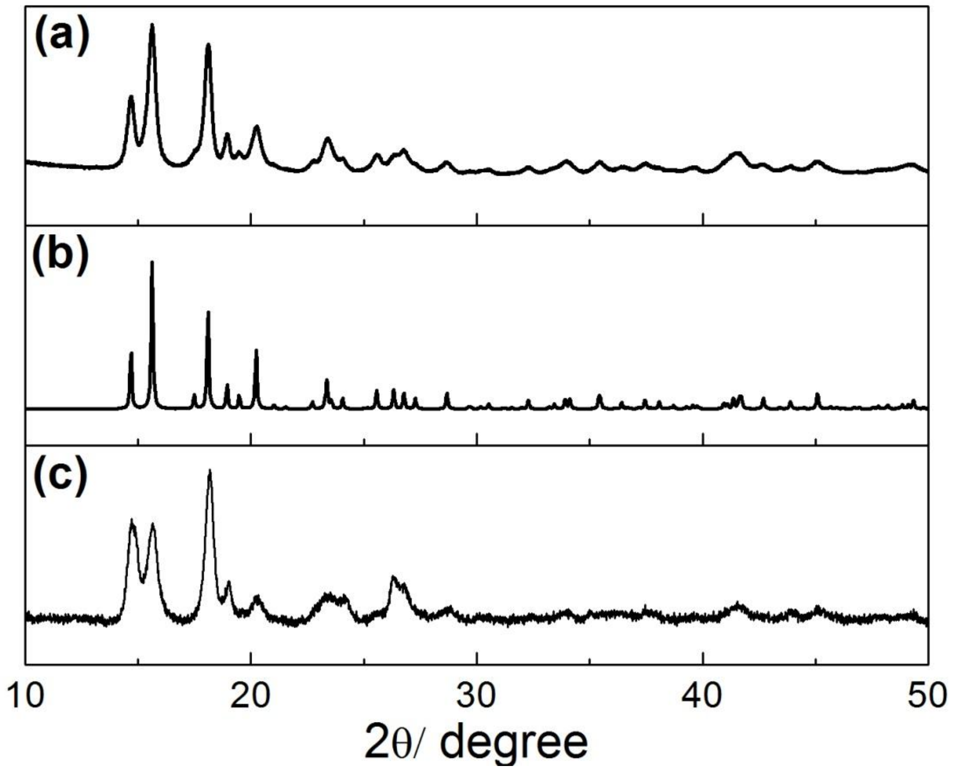

Fig. S10 XRD patterns of (a) nanosheet-shaped [Zn(C_2O_4)(EG)], (b) single crystal [Zn(C_2O_4)(EG)], and (c) nanosheet-shaped [Co(C_2O_4)(EG)] : the synthesized-[Co(C_2O_4)(EG)] has the same XRD pattern as the synthesized-[Zn(C_2O_4)(EG)] and the different intensities are attributed to different crystal size [S3].

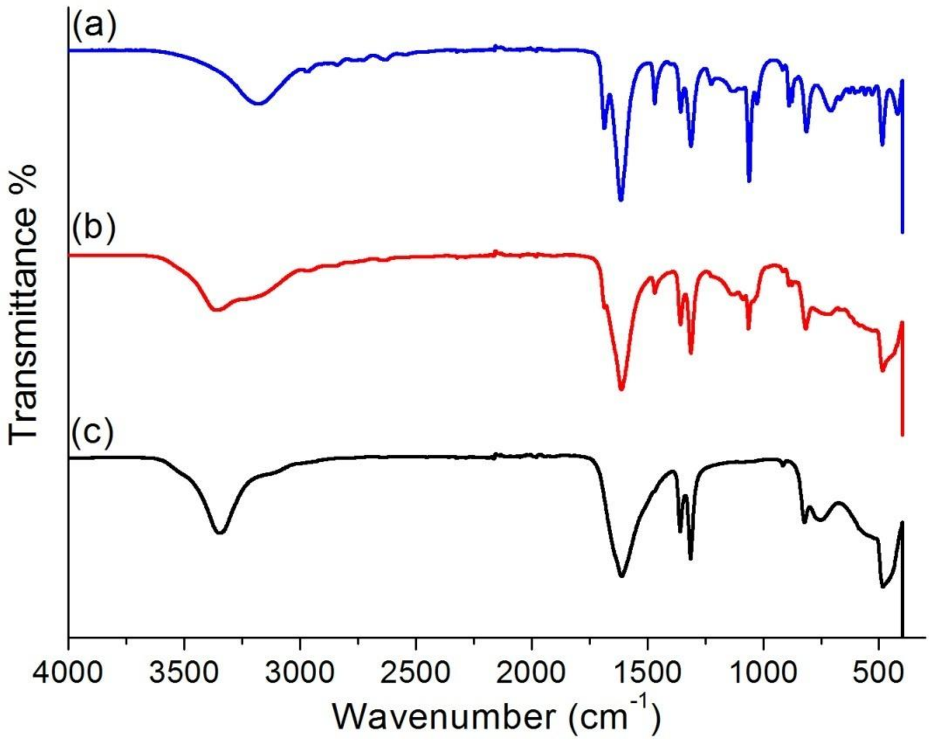

(a)	(b)	(c)	Assignment
	3363	3348	O-H stretch
3180	3180		
2800~3000	2800~3000		C-H stretch (alkane)
1686	1686		C=O stretch
1615	1614	1612	
1470	1469		C-H bend (alkane)
1357	1357	1358	symmetric and asymmetric O-C-O
1312	1313	1314	
1061	1063		O-C stretch
814	817	822	asymmetric O-C-O band
486	485	484	Co-O stretch

Fig. S11 FT-IR spectra of (a) nanosheet-shaped $[Co(C_2O_4)(EG)]$ complex, (b) nanorod-shaped $[Co(C_2O_4)(EG)_x(OH_2)_y]$ complex, and (c) nanorod-shaped $[Co(C_2O_4)(OH_2)_2]$ complex.

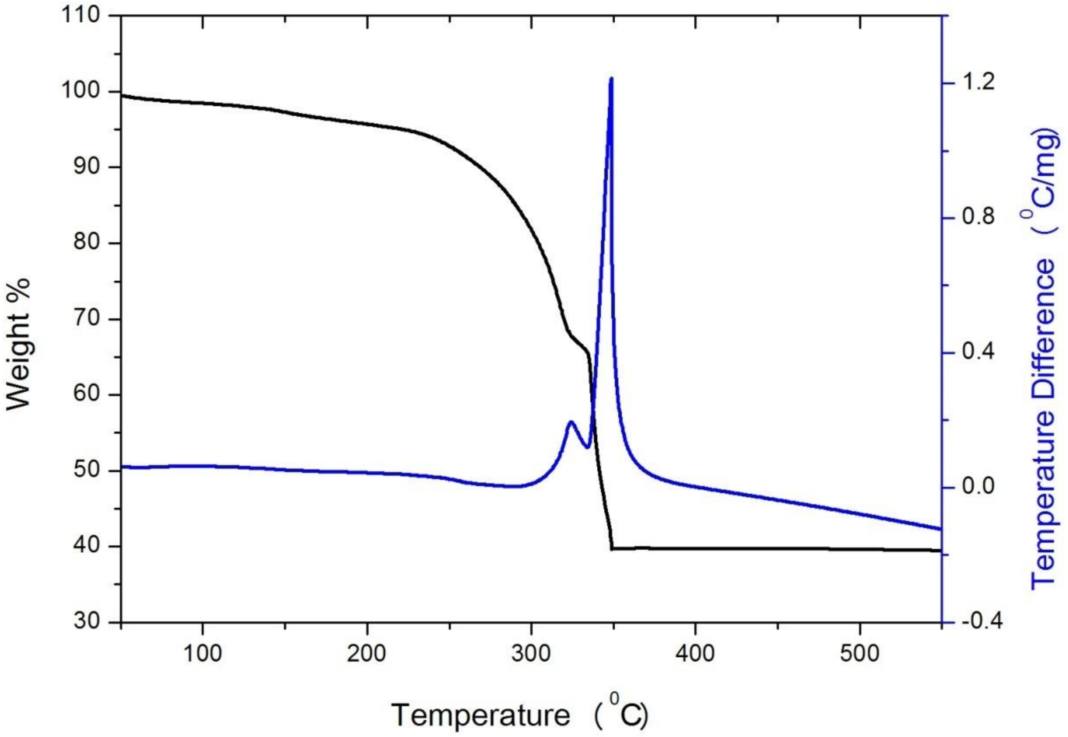

Temperature (℃)	Weight loss		Assginment
	Theoretical (%)	Observed (%)	
~328	29.70	30.20	EG
328~360	38.40	38.43	Co_3O_4

Fig. S12 TGA-DTA analysis of nanosheet-shaped [$Co(C_2O_4)(EG)$] complex (Black: TGA, Blue: DTA).

M. SEM, XRD, HR-TEM, FTT and TGA analyses of nanorod-shaped [Co(C_2O_4)(EG)$_x$(OH$_2$)$_y$] and nanorod-shaped [Co(C_2O_4)(OH$_2$)$_2$]

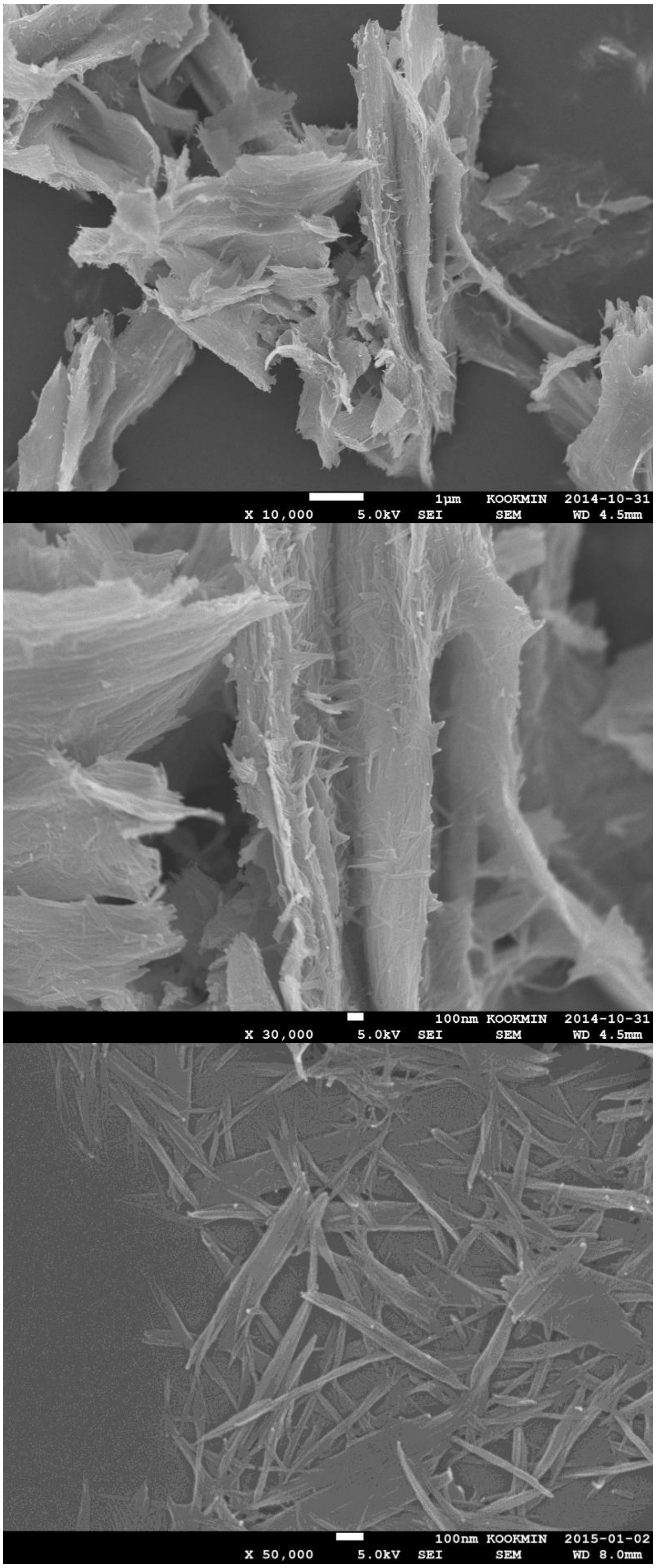

Fig. S13 SEM images of nanorod-shaped [Co(C_2O_4)(EG)$_x$(OH$_2$)$_y$] and [Co(C_2O_4)(OH$_2$)$_2$] complex.

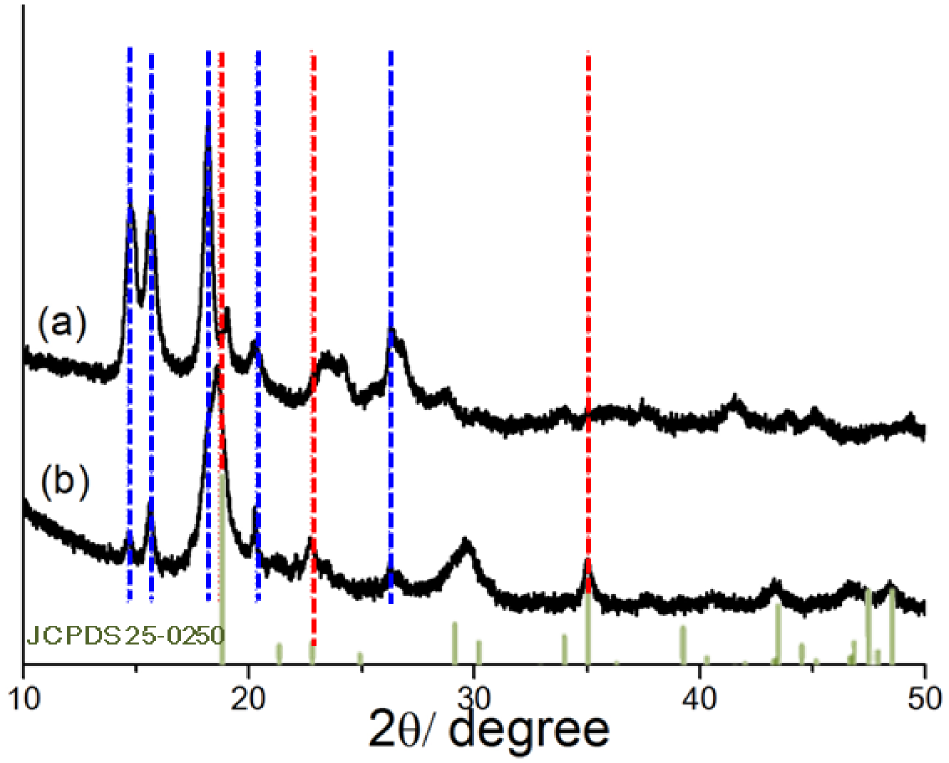

Fig. S14 XRD patterns of (a) nanosheet-shaped [Co(C_2O_4)(EG)], (b) nanorod-shaped [Co(C_2O_4)($EG)_x$($OH_2)_y$], and the well-known [Co(C_2O_4)($OH_2)_2$] (JCPDS 25-0250): Blue lines indicate [Co(C_2O_4)(EG)] complex and Red lines indicate [Co(C_2O_4)($OH_2)_2$] complex. **Fig. S13** indicates that adding OH_2 to [Co(C_2O_4)(EG)] renders the ligands, EG, switched to OH_2.

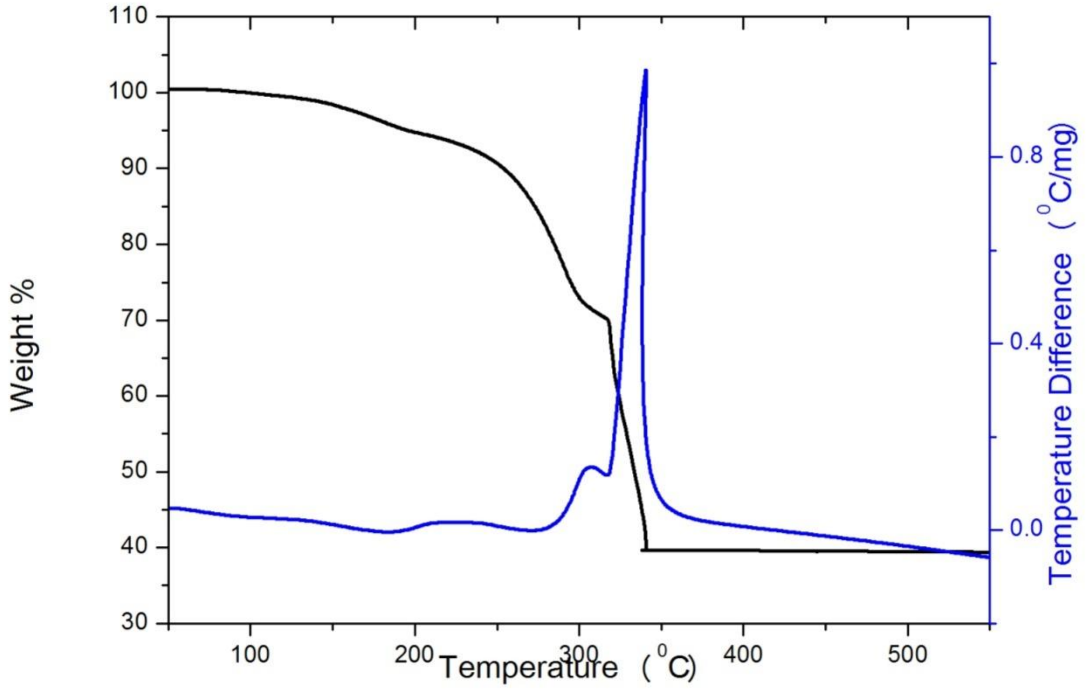

Temperature (℃)	Endothermic peak (℃)	Weight loss Observed (%)	Assginment	Ratio
125 ~ 215	183	4.85	OH_2	1
237 ~ 306	271	22.58	EG	1.35
		39.67	Co_3O_4	-

Fig. S15 TGA-DTA analysis of nanorod-shaped $[Co(C_2O_4)(EG)_x(OH_2)_y]$ complex (Black: TGA, Blue: DTA).

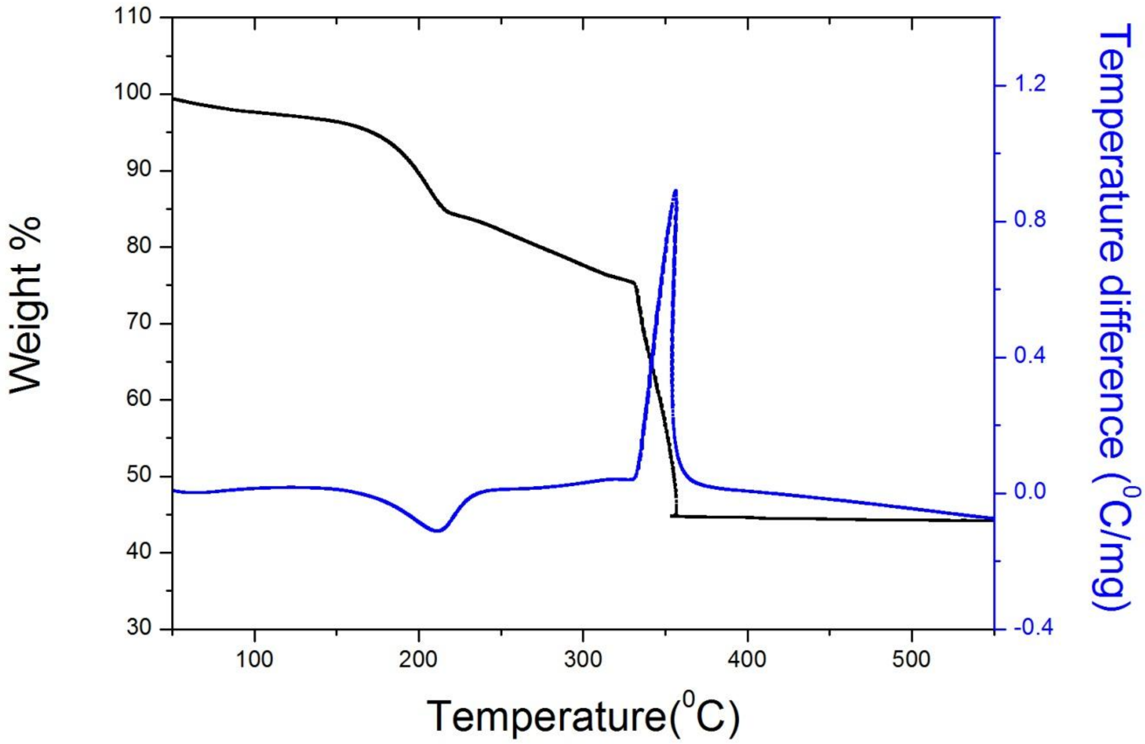

Temperature (℃)	Endothermic peak (℃)	Weight loss		Assginment
		Theoretical (%)	Observed (%)	
143 ~ 231	180	19.69	18.64	OH_2
		43.87	44.32	Co_3O_4

Fig. S16 TGA-DTA analysis of nanorod-shaped $[Co(C_2O_4)(OH_2)_2]$ complex (Black: TGA, Blue: DTA).

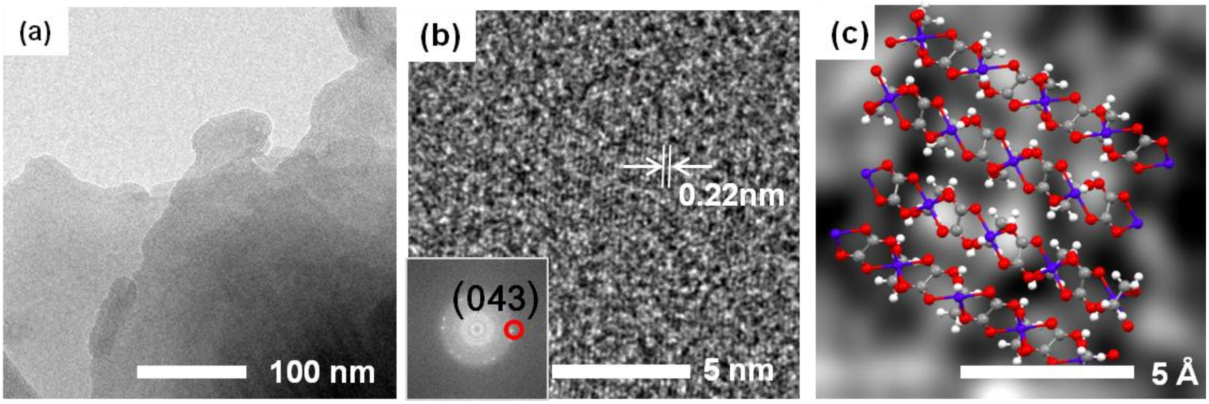

Fig. S17 (a) TEM, (b) HR-TEM, (b, inset) FTT images of nanosheet-shaped [Co(C_2O_4)(EG)], and (c) the [Co(C_2O_4](EG)] molecular structure is overlaid on the image's (043) surface obtained by HR-TEM (white : hydrogen, grey : carbon, red : oxygen, and purple : cobalt). The structure consisting the surface is interacted by the H-bond between EG and Oxalate in the same way as [Zn(C_2O_4)(EG)].

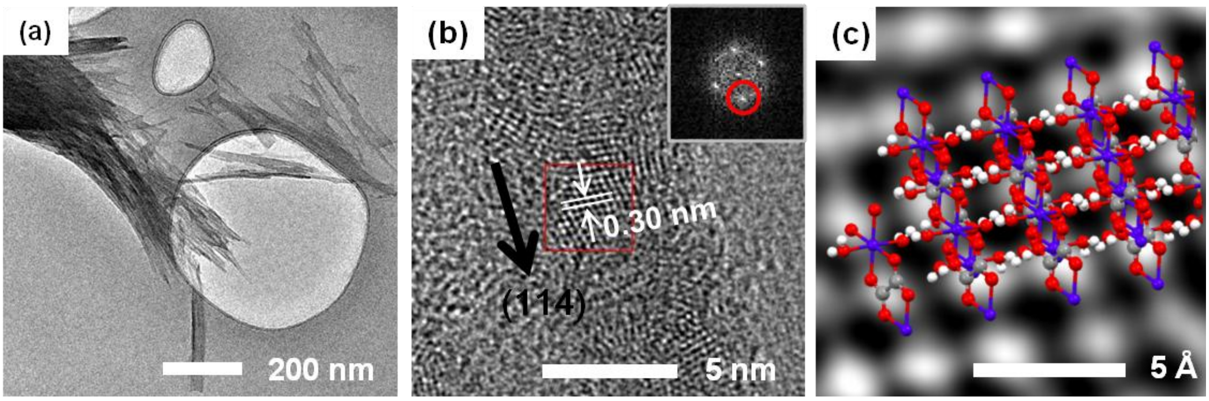

Fig. S18 (a) TEM, (b) HR-TEM, (b, inset) FTT images and (c) (144) face's molecular structure of nanorod-shaped [Co(C_2O_4)(OH_2)$_2$] (grey : carbon, red : oxygen, purple : cobalt, and hydrogen atoms are omitted for clarity). The structure consisting the surface is interacted by the H-bond between OH_2 and Oxalate in the same way as [Zn(C_2O_4)(OH_2)$_2$].

N. SEM, HR-TEM, FTT images and XRD graphs of the prepared-ZnO samples

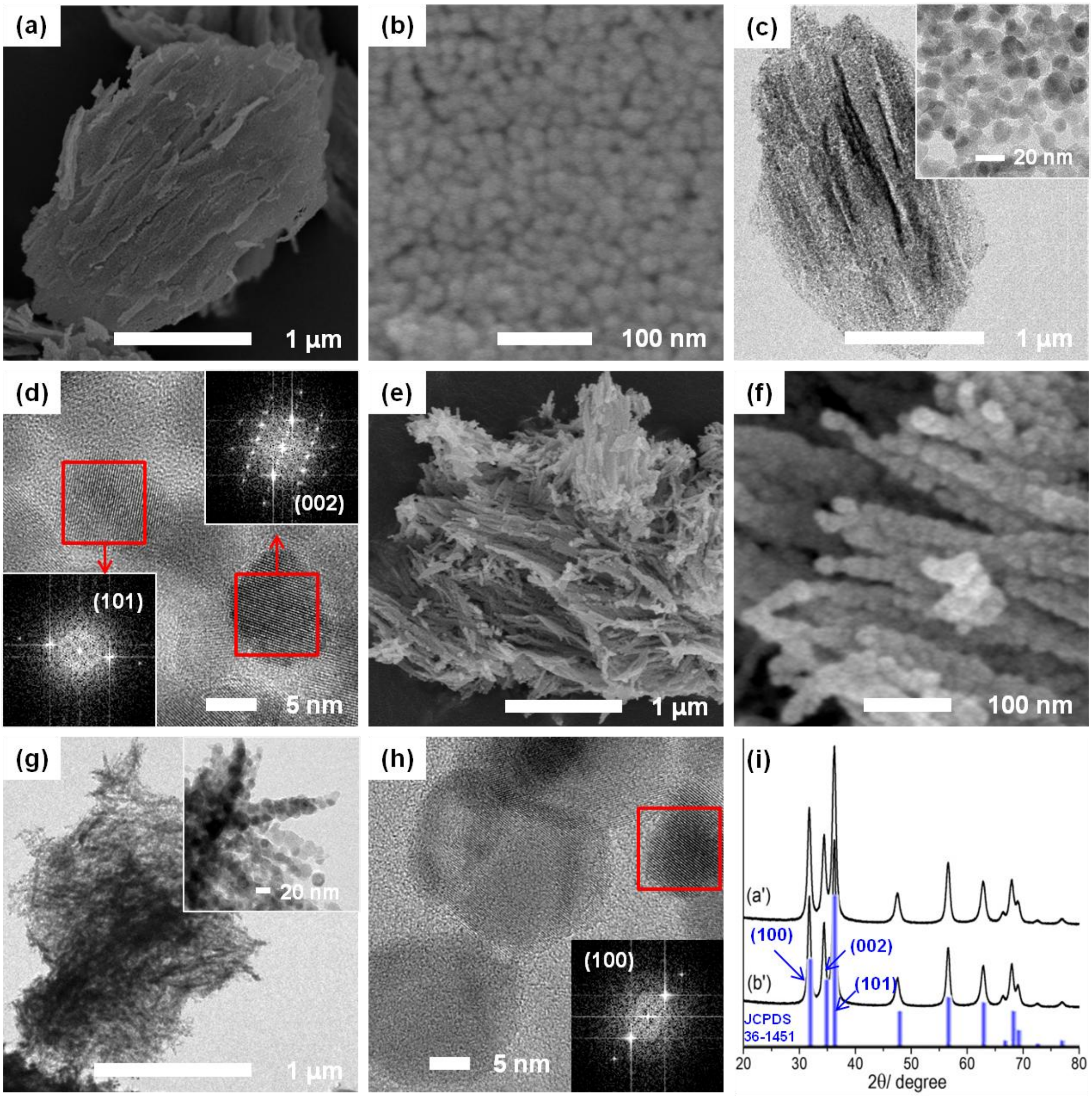

Fig. S19 (a and b) SEM images, (c and d) TEM, HR-TEM and FTT-TEM images of nanosheet-shaped ZnO from [Zn(C_2O_4)(EG)] precursor, (e and f) SEM images, (g and h) TEM, HR-TEM and FTT-TEM images of nanorod-shaped ZnO from [Zn(C_2O_4)(OH_2)$_2$] precursor, and (i) XRD patterns of ZnO samples; (a') nanosheet-shaped ZnO from [Zn(C_2O_4)(EG)] precursor, (b') nanorod-shaped ZnO from [Zn(C_2O_4)(OH_2)$_2$] precursor and JCPDS No. 36-1451 of ZnO.

O. SEM, HR-TEM, FTT images and XRD graphs of the prepared-Co_3O_4

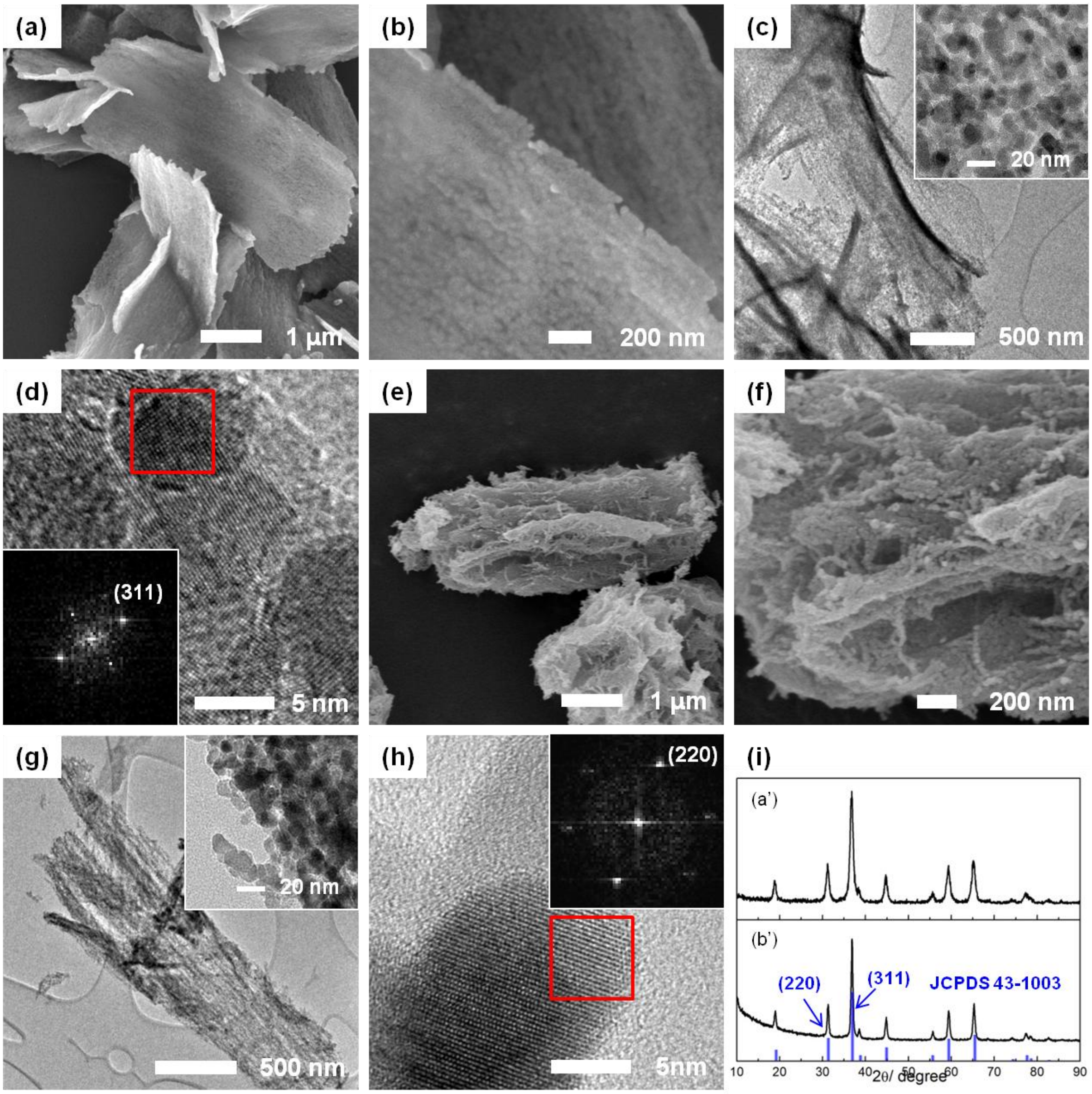

Fig. S20 (a and b) SEM images, (c and d) TEM, HR-TEM and FTT-TEM images of nanosheet-shaped Co_3O_4 from [Co(C_2O_4)(EG)] precursor, (e and f) SEM images, (g and h) TEM, HR-TEM and FTT-TEM images of nanorod-shaped Co_3O_4 from [Co(C_2O_4)(OH_2)$_2$] precursor, and (i) XRD patterns of Co_3O_4 samples; (a') nanosheet-shaped Co_3O_4 from [Co(C_2O_4)(EG)] precursor, (b') nanorod-shaped Co_3O_4 from [Co(C_2O_4)(OH_2)$_2$] precursor and JCPDS No. 43-1003 of Co_3O_4.

P. BET analysis of the prepared-metal oxide samples

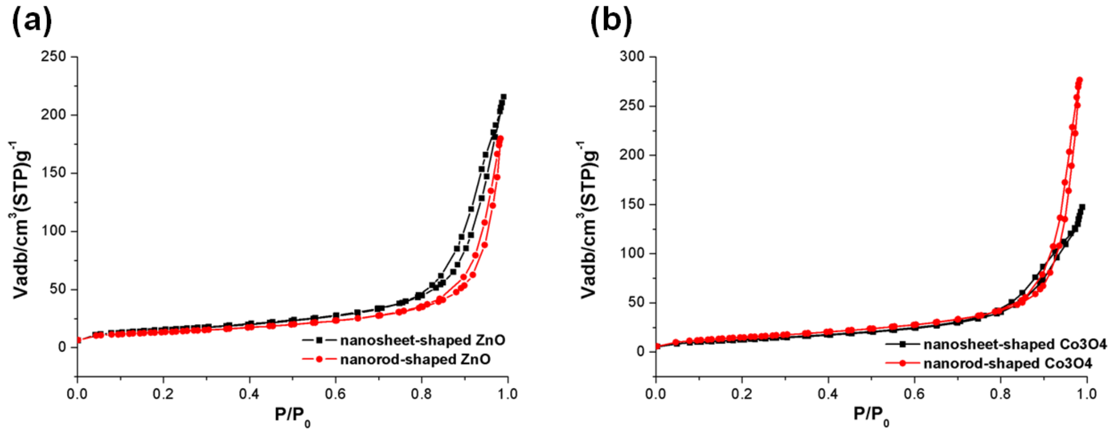

Fig. S21 N_2 adsorption-desorption isotherms of samples (a) nanosheet-shaped ZnO and nanorod-shaped ZnO, and (b) nanosheet-shaped Co_3O_4 and nanorod-shaped Co_3O_4.

References:

[S1] Tan, Z.-D., Tan, F.-J., Tan, B. & Zhang, C.-M. catena-Poly[(ethanediol-κ^2*O*,*O*')zinc]-υ-oxalato-κ^4*O*^{1},*O*^{2}:*O*$^{1'}$,*O*$^{2'}$]. *Acta Cryst.* **E68**, m868 (2012).

[S2] Giester, G. Syntheses and crystal structures of $Co_3(C_2O_4)(SeO_3)_2$ and $Zn(C_2O_4)\cdot 2H_2O$. *Z. Kristallogr* **212**, 720-723 (1997).

[S3] Inoue, M. & Hirasawa, I. The relationship between crystal morphology and XRD peak intensity on $CaSO_4\cdot 2H_2O$. *J. Cryst. Growth.* **380**, 169-175 (2013).
